# Retraction: Inactivation of MAP kinase signalling in Myc Transformed Cells and Rescue by LiCl inhibition of GSK3

**DOI:** 10.1186/1476-4598-4-17

**Published:** 2005-05-06

**Authors:** Osama Al-Assar, Dorothy H Crouch

**Affiliations:** 1 Biomedical Research Centre, University of Dundee, Ninewells Hospital and Medical School, Dundee DD1 9SY, UK; 2 Institute for Cancer Studies, Division of Genomic Medicine, Medical School, University of Sheffield, Beech Hill Road, Sheffield S10 2RX, UK

## Abstract

The corresponding author submitted this article [1] to Molecular Cancer on the assumption that the co-author had agreed to the submission. Since this is not the case, the authors are retracting the article. The corresponding author is deeply sorry for any inconvenience this may have caused to the editorial and publishing staff. An apology is also extended to the readers.
